# Olive oil bioactives protect pigs against experimentally-induced chronic inflammation independently of alterations in gut microbiota

**DOI:** 10.1371/journal.pone.0174239

**Published:** 2017-03-27

**Authors:** Martin Liehr, Alessandro Mereu, Jose Javier Pastor, Jose Carlos Quintela, Stefanie Staats, Gerald Rimbach, Ignacio Rodolfo Ipharraguerre

**Affiliations:** 1 Institute of Human Nutrition and Food Science, University of Kiel, Kiel, Germany; 2 Lucta S.A., Montornés del Vallés, Barcelona, Spain; 3 ProNutra Solutions SL, Madrid, Spain; Wageningen University, NETHERLANDS

## Abstract

Subclinical chronic inflammation (SCI) is associated with impaired animal growth. Previous work has demonstrated that olive-derived plant bioactives exhibit anti-inflammatory properties that could possibly counteract the growth-depressing effects of SCI. To test this hypothesis and define the underlying mechanism, we conducted a 30-day study in which piglets fed an olive-oil bioactive extract (OBE) and their control counterparts (C+) were injected repeatedly during the last 10 days of the study with increasing doses of *Escherichia coli* lipopolysaccharides (LPS) to induce SCI. A third group of piglets remained untreated throughout the study and served as a negative control (C-). In C+ pigs, SCI increased the circulating concentration of interleukin 1 beta (*p* < 0.001) and decreased feed ingestion (*p* < 0.05) and weight gain (*p* < 0.05). These responses were not observed in OBE animals. Although intestinal inflammation and colonic microbial ecology was not altered by treatments, OBE enhanced ileal mRNA abundance of tight and adherens junctional proteins (*p* < 0.05) and plasma recovery of mannitol (*p* < 0.05) compared with C+ and C-. In line with these findings, OBE improved transepithelial electrical resistance (*p* < 0.01) in TNF-α-challenged Caco-2/TC-7 cells, and repressed the production of inflammatory cytokines (*p* < 0.05) in LPS-stimulated macrophages. In summary, this work demonstrates that OBE attenuates the suppressing effect of SCI on animal growth through a mechanism that appears to involve improvements in intestinal integrity unrelated to alterations in gut microbial ecology and function.

## Introduction

Inflammation is a protective mechanism of higher organisms that aids in coping with stressors and harmful environmental stimuli [1]. Despite being tremendously complex and involving a variety of immune cells, blood vessels, and molecular mediators, inflammatory processes can be distinguished into two somewhat different types [2; 3] termed herein clinical and subclinical chronic inflammation (SCI). In contrast to clinical inflammation resulting for instance from injury or bacterial infection, SCI appears as a much milder but persistent response of the host’s immune system [4].

Initial steps following immune system activation involve the release of pro-inflammatory cytokines to counteract migration and spreading of potential antigens. Tumor necrosis factor alpha (TNF-α) and interleukin 1 beta (IL1B) belong to the group of pro-inflammatory first order cytokines, released in the early stages of inflammation [5]. Immediate effects of these cytokines are numerous and include fever as well as recruitment, activation and differentiation of immune cells at the site of ongoing inflammation [6; 7; 8]. Usually the period of cytokine action is tightly regulated through various control mechanisms and thereby strictly limited to the initial phases of the inflammation process [9]. However, under certain circumstances elevated plasma levels of pro-inflammatory mediators are maintained throughout a prolonged period ultimately manifesting as SCI. In addition to mediate tissue specific disorders, SCI can cause significant metabolic alterations in the organism as a consequence of cytokine-induced anorexia. Decreased feed intake along with a shift in nutrient utilization from maintenance, growth or processes that involve anabolic conditions towards immune defense bring the metabolic state of the animal into catabolism [10].

Even though there are several ways to treat inflammation with high efficiency (e.g., through administration of corticosteroids or non-steroidal anti-inflammatory drugs), especially long term application of these interventions usually comes along with numerous side effects. Fruits and leaves of the olive oil tree (*Olea europaea* L.) contain significant amounts of hydrophilic and lipophilic bioactives including flavones, phenolic acids, phenolic alcohols, secoiridoids and hydroxycinnamic acid derivates [11; 12; 13; 14; 15]. As a result of their anti-inflammatory, antioxidant, and antimicrobial actions, olive-derived plant bioactives have been shown to cause several beneficial effects under pathological conditions [16; 17; 18; 19; 20], which renders them promising feed additives.

We postulated that dietary supplementation with an olive-oil extract enriched in olive bioactives (OBE) may contribute to counteract SCI-induced growth depression. To test this hypothesis we used a model of experimentally induced chronic inflammation in weaned pigs fed a diet supplemented with OBE. An emerging body of evidence indicates that plant bioactives, including those from *Olea europaea*, exert partly their beneficial actions indirectly *via* modulation of or modification by the intestinal microbiota [21]. Consequently, we also investigated the impact that OBE has on gut microbiota of control and SCI-challenged pigs. Additionally, a series of cell culture studies were conducted to delineate mechanistic components of the *in vivo* mode of action of OBE.

## Materials and methods

### Animals and housing

A total of 31 male piglets (Landrace X Landrace X Pietrain) were housed in the nursing facilities of Lucta S.A. (Girona, Spain). Piglets were weaned at 25.0 ± 1.0 d of age weighing 7.1 ± 1.2 kg. Animals were housed into individual pens (0.35 m^2^/pen) equipped with fully slatted plastic floor plus a nipple drinker and a feeder with *ad libitum* access to water and a commercial non-medicated pre-starter diet (Table 1). Room temperature was thermostatically set at 30°C, and the daily lighting photoperiod lasted 12 h (from 08:00 a.m. to 08:00 p.m.). Body weight (BW) was measured at weaning (d 1) and d 7, 14, 19 and 29 after weaning. Feed consumption was measured weekly until d 19 and daily from d 20 to d 29. Health status of individual animals was assessed daily according to the Guidelines on the recognition of pain, distress and discomfort (Table 2, adapted from Morton and Griffiths, 1985 [22]). In case of death, animals were disposed promptly by a commercial rendering service (Sereca Bio, SL; Spain). A post-mortem examination was performed to provide important animal health information to prevent further losses.

**Table 1:** Ingredients and chemical composition of the experimental diet.

Ingredient (g/kg air-dried diet)	
Corn	320
Full fat soy	200
Wheat	136
Soybean meal concentrate (56% CP)	120
Lactose	86
Barley	64
Soybean oil	34
Monocalcium phosphate	12.7
Calcium carbonate	10.8
Salt	3
L-Lysine-HCl	5
Trace elements and vitamin premix ^a^	4
DL-Methionine	2.2
L-Threonine	2
L-Tryptophan	0.3
***Calculated analysis (% or as specified)***	
Crude protein (N x 6.25)	19.4
Digestible amino acids^b^	
Lysine	1.38
Methionine	0.50
Methionine + Cysteine	0.83
Threonine	0.90
Tryptophan	0.26

^a^The mineral and vitamin premix (TEGASA, Barcelona, Spain) provided the following per kg of diet: vitamin A 10,000 IU; vitamin D3 2000 IU; vitamin E 25 mg; vitamin B1 1.5 mg; vitamin B2 3.5 mg; vitamin B6 2.4 mg; vitamin B12 20 μg; vitamin K3 1.5 mg; calcium pantothenate 14 mg; nicotinic acid 20 mg; folic acid 0.5 mg; biotin 50 μg; iron 120 mg; iodine 0.75 mg; cobalt 0.6 mg; copper 150 mg; manganese 60 mg; zinc 110 mg; selenium 0.37 mg

^b^Ileal standardized digestibility

**Table 2:** Guidelines on the recognition of pain, distress and discomfort.

Weight	Appearance	Feeding behaviour	Response to handling	Clinical parameters
0: normal weight.	0: no diarrhea, normal skin, normal walk and no injuries.	0: normal food and water intake.	0: normal pattern behaviour.	0: Checking of temperature, respiratory and digestive systems are ok.
1: light losses (less than 10% of BW)	1: any signs of disease (diarrhea or respiratory problem), lameness, any injuries and furry piglet.	1: reduced food and water intake.	1: signs of pain to handling (heavy vocalisations and abnormal locomotion)	1: fever, abnormal breathing pattern, nasal discharge, abnormal faeces, decrease in BW and abnormal locomotion.
2: heavy losses (more than 20% of BW). Underweight animals.	2: appear sleepy, less active, with a pinched abdomen and sunken eyes	2: no feeding behaviour.	2: no reactivity to handling.	2: reduced breathing pattern, no activity, and hypothermia.

Piglets would have been sacrificed when all evaluations are in level number 2.

### Experimental design and treatments

On d 1, animals were divided into 3 groups balanced by BW and assigned to 3 experimental treatments that were negative control (C-, n = 10), positive control (C+, n = 11) offered same feed as C-, and OBE (OBE, n = 10) offered same feed as C- but supplemented with 500 mg/kg of an olive-oil extract enriched in polyphenols and triterpenic acids. Starting on d 20, pigs received an intraperitoneal (i.p.) injection every 72 h for a total of 3 injections/pig. Pigs in the C- group were injected with saline, whereas C+ and OBE pigs were administered LPS from *E*. *coli* (serotype O55:B5; Sigma-Aldrich, Madrid, Spain) reconstituted in saline at increasing doses (60, 66, 72 μg/kg BW) to induce SCI. The doses of LPS were established in line with previous work [23]. On d 28 and 29 after 2 h of fasting, pigs received an additional i.p. injection of LPS (78 μg/kg BW) and 3 h later were sacrificed for sample collection.

### Olive-oil bioactive extract

The olive-oil bioactive extract (Lucta S.A., Barcelona, Spain) was obtained by a proprietary process described in patent US8361518B2 [24]. Pomace olive-oil was filtrated and bioactive compounds present in the solid fraction were extracted and purified with ethanol. The final product (OBE) was dissolved in methanol (5 mg/mL) and quantified by HPLC following the chromatographic conditions described by Romero *et al*. [25] for triterpenic acids and by Mulinacci *et al*. [26] for hydroxytyrosol. Finally, OBE was standardized to 10% maslinic acid, 4% oleanolic acid and 2% hydroxytyrosol (16% on DM basis).

### Collection of plasma and intestinal samples

Three h after the final LPS challenge, blood samples were collected *via* jugular venipuncture in tubes containing EDTA and aprotinin (BD Vacutainer^®^), held in ice-cold water for 30 min, centrifuged at 2,000×g for 10 min, and stored frozen at -80°C until analyses. Immediately after bleeding, pigs were stunned with captive bolt and sacrificed *via* exsanguination. The abdomen was opened, the intestine was removed and the ileum (from the first Peyer’s patch to the ileocecal valve) was dissected. A 5 cm segment was removed from the midsection of the ileum, opened longitudinally and flushed with saline. The mucosa was scraped, placed in RNA*later* (Applied Biosystems *via* Thermo Fisher Scientific, Darmstadt, Germany) and then stored at -80°C until gene expression analysis. In addition, the content of a 10-cm section of the ascendant colon proximal to the ileo-cecal valve was placed in 50-mL cryovials, snap-frozen, and stored at -80°C until microbiota analysis.

### Determination of plasma cytokines and acute-phase proteins

Plasma concentrations of IL1B and the acute-phase protein (APP) pigMAP were analyzed by ELISA (Cusabio, Wuhan, China). All experimental steps were executed as mentioned in the manufactures protocol. In brief, diluted samples and standards were applied to the microwell assay plate incubated for 2 h at 37°C to allow bounding of target proteins. Subsequently, samples were incubated with biotin-conjugated antibody for 1 h at 37°C and after multiple washings were treated with horseradish peroxidase (HRP) conjugated avidin. Following repeated washings to remove unbound avidin, 3, 3’, 5, 5’-tetramethylbenzidin (TMB) substrate solution was added and color intensity was determined photometrically at 450nm. Values were calculated *via* standard curve (CurveExpert, version 1.4, Madison, United States of America).

### Analysis of intestinal microbiota

Samples of colonic content were thawed and immediately processed to isolate bacterial DNA to assess the microbiome profile by massive sequencing of the hypervariable regions of the 16S rRNA gene. Briefly, amplicons of the V1-V2 16S rRNA region were amplified with barcoded forward primer F27 and reverse primer R338, with sequencing adaptors at the 5′ end. Concentration and quality were determined using Agilent Bioanalyzer 2100 for each amplicon. Samples were sequenced on an Ion Torrent Personal Genome Machine (PGM) with the Ion 318 Chip Kit v2 (Life Technologies) under manufacturer’s conditions.

### Assessment of intestinal permeability

Intestinal permeability was assessed *in vivo* on d 28 and 29 after weaning. Eight pigs per treatment (representative of the median BW of each treatment) were fasted for 2 h and subsequently sedated with a mixture of xylazine (1.5 mg/kg BW) and ketamine (11 mg/kg BW) administered intramuscularly (i.m.) in order to minimize handling stress. After 10 min, animals were intragastrically dosed (gastroduodenal feeding tube, Levin type; VEC Medical) with a marker solution containing 0.15 g mannitol/kg BW (Sigma-Aldrich, Madrid, Spain) and 0.1 g Co-EDTA/kg BW [27] dissolved in 15 mL deionized water and immediately after were challenged with LPS as previously described. Blood samples were collected by jugular venipuncture 1 h after oral infusion of permeability markers into 5 mL evacuated fluoride/K-oxalate glucose blood collection tubes (BD vacutainer, Madrid, Spain). Plasma mannitol was determined by ultra-high performance liquid-chromatography mass-spectrometry (Xevo G2 TOF, Waters) as previously described [28]. The plasma concentration of cobalt was determined by atomic absorption spectroscopy [27].

### Ethics statement

The study with pigs was carried out according to the recommendations of the *Guide for the Care and Use of Laboratory Animals of the National Institutes of Health*, making all possible efforts to minimize animal suffering. All experimental procedures were approved by the Laboratory Animal Care Advisory Committee of the Faculty of Veterinary Sciences of the Universitat Autónoma de Barcelona, Spain.

### Cell culture studies

Caco-2/TC-7 [29] human colon carcinoma epithelial cells and RAW 264.7 [30] murine macrophages were maintained in Dulbecco’s Modified Eagle Medium (DMEM) containing 4.5 g/L glucose, 4 mmol/L L-glutamine, 1 mmol/L sodium pyruvate, 100 U/mL penicillin, 100 μg/mL streptomycin and fetal calf serum (FCS, 20 and 10% (v/v), respectively, Gibco *via* Thermo Fisher Scientific, Darmstadt, Germany). Both cell lines were grown in 5% CO_2_/95% air at 37°C in a humidified atmosphere. For sub-culturing, cells were detached with 0.05% trypsin and 0.02% EDTA (Caco-2/TC-7) or were scraped off (RAW 264.7). Cell culture reagents were purchased from PAN-Biotech GmbH (Aidenbach, Germany). Cell culture plastic labware was obtained from Sarstedt (Nuembrecht, Germany) except when otherwise mentioned.

### Assessment of cytotoxicity

Determination of treatment-related cytotoxicity was performed with the neutral red assay as described previously [31]. Both, Caco-2/TC-7 and RAW 264.7 cells were pre-cultured for 24 h before being incubated with increasing concentrations of OBE (1–100 μg/mL) for 24 or 48 h. Ethanol (10% v/v) was used as positive control to induce cell death. Following treatment medium was replaced with culture medium containing 50 μg/mL neutral red dye (Carl Roth, Karlsruhe, Germany) and incubated for 2 h. Cells were washed once with phosphate buffered saline (PBS, Gibco *via* Thermo Fisher Scientific, Darmstadt, Germany) and incubated with neutral red extraction buffer (50% ethanol, 49% double distilled water, 1% glacial acetic acid) for 15 min on a shaking platform. The absorbance of neutral red dye was measured at 450 nm and viability of the compound-treated cells was calculated as percentage absorbance of the vehicle treated cells for each treatment.

### Determination of inflammatory response *in vitro*

To investigate cytokine gene expression *in vitro* murine RAW 264.7 macrophages were seeded at an initial density of 4.0x10^6^cells/cm^2^ into polysterol cell culture plates. After 24 h of pre-cultivation cells were incubated with the highest non-toxic concentration of OBE (50 and 25 μg/mL) for another 24 h and subsequently stimulated with *Salmonella enterica*-derived LPS (10 ng/mL, Sigma-Aldrich, Taufkirchen, Germany) for 6 h to induce macrophage activation. Cells were washed once with ice cold PBS and subsequently prepared for RNA isolation (see below).

### Determination of intestinal barrier function *in vitro*

Caco-2/TC-7 cells spontaneously start to differentiate after reaching confluence and develop a polarized small intestinal enterocyte-like phenotype [32] thus providing a suitable model to quantify epithelial barrier function *in vitro* [33]. For the determination of the transepithelial electrical resistance (TEER) Caco-2/TC-7 cells were grown on permeable filters (Corning, Inc., Corning, NY, USA, 0.4 mm pore size) at an initial density of 9.0x10^4^ cells/cm^2^. After 4 d of pre-cultivation, confluent monolayers were apically treated with the highest non-toxic concentration of OBE (OBE; 100 μg/mL) for a further 5 d. TEER was measured with a chopstick electrode (Millicell ERS-2V-Ohm Meter, Darmstadt, Germany) on d 0, 2, 4 and 5 of OBE treatment to determine barrier tightness. To induce barrier disruption, TNF-α (100 ng/mL; Immunotools, Friesoythe, Germany) was added to the basolateral compartment for 24 h prior to final TEER measurement. To eliminate temperature-related changes in TEER cells were equilibrated for 15 min at room temperatures before each readout. The numerical calculation of TEER was done as follows:

$$\begin{array}{l} TEER\;\left(\frac{\Omega }{cm^{2}}\right)=\\ \left(resistance\;of\;treated\;cells\;\left(\Omega \right)\;-\;resistance\;of\;blank\;well\;\left(\Omega \right)\right)\;\times \\ effective\;membrane\;area\;\left(cm^{2}\right)\;normalized\;to\;the\;control\;\left(vehicle\;treated\;cells\right) \end{array}$$

### Determination of cytokine and junctional protein mRNA levels *in vitro* and in porcine tissue samples (qRT-PCR analyses)

Total RNA from ileal tissue and murine macrophages was isolated with peqGOLDTriFast^™^ (PEQLAB Biotechnologie GmbH, Erlangen, Germany) following manufacturer’s instructions. To measure junctional protein mRNA levels in porcine samples, tissues were homogenized with a TissueLyser II (Qiagen, Hilden, Germany) and total RNA was extracted with TriFast reagent according to the manufacturer’s protocol. RT-PCR primers were designed using PRIMER 3 software (v. 0.4.0) and were purchased from MWG Eurofins (Ebersberg, Germany). Gene expression levels in tissues and cells were determined by quantitative RT-PCR using the SensiFAST^™^ SYBR^®^ No-ROX One-Step Kit (Bioline, Luckenwalde, Germany) with SybrGreen detection in a Rotorgene 6000 cycler (Corbett Life Science, Sydney, Australia). Relative mRNA quantification was calculated using a standard curve. Target gene expression (Table 3) was normalized to the expression of the housekeeping gene GAPDH or the mean of GAPDH and beta-actin.

**Table 3:** Oligonucleotide sequences used to determine target gene expression.

Target gene	Gene ID	Nucleotide sequence	Annealing temperature (°C)
*Sus scrofa*			
GAPDH	396823	GTCGGTTGTGGATCTGACCT	60
		TCACAGGACACAACCTGGTC	
CDH1	100048953	TGAAGAAGGAGGTGGAGAAG	57
		GTGCCACATCATTACGAGTC	
OCLN	397236	GGCCATATCCAGAGTCTTCG	60
		ACGCCTCCAAGTTACCACTG	
ZO-1	396567	GGCCATATCCAGAGTCTTCG	57
		ACGCCTCCAAGTTACCACTG	
IL1B	397122	CCTCTCCAGCCAGTCTTC	57
		GGGTGCAGCACTTCATCTCT	
iNOS	396859	GTCCAGCGCTACAACATCCT	57
		TCCATGATGGTCACGTTCTG	
TNF-α	397086	CTCTTCTCCTTCCTCCTGGT	57
		ACGATGATCTGAGTCCTTGG	
*Mus musculus*			
IL1B	16176	CAG GCA GGC AGT ATC ACT CA	55
		AGC TCA TAT GGG TCC GAC AG	
iNOS	18126	GGCAGCCTGTGAGACCTTTG	58
		GCATTGGAAGTGAAGCGTTTC	
Mip1a	20302	CCT CTG TCA CCT GCT CAA CA	58
		GAT GAA TTG GCG TGG AAT CT	
ACTB	11461	GAC AGG ATG CAG AAG AGA TTA CT	55
		TGA TCC ACA TCT GCT GGA AGG T	
GAPDH	14433	CCG CAT CTT CTT GTG CAG T	57
		GGC AAC AAT CTC CAC TTT GC	

ACTB, beta-actin; CDH1, E-cadherin; GAPDH, glyceraldehyde-3-phosphate dehydrogenase; IL1B, interleukin 1 beta; Mip1a, macrophage inflammatory protein 1 alpha; iNOS, nitric oxide synthase 2; OCLN, occludin; TNF-α, tumor necrosis factor alpha; ZO-1, zonula occludens 1

### Statistical analysis

In view of the objectives of the study, parameters for animal performance including feed consumption, body weight gain, and efficiency of feed conversion were analyzed from weaning until the end of nursing phase (d 29) by using a mixed-effect model with repeated measures in time. In the model, the pig nested within the treatment entered as random variable and treatment, time and their two-way interaction were considered as fixed effects. The smallest value for the Akaike’s information criterion was used to identify the most appropriate covariance structure. The same model but without repeated measures was used to analyze plasma concentrations of permeability markers (cobalt and mannitol), cytokines, and APP. Model diagnostics included testing for a normal distribution of the error residuals and homogeneity of variance. Gene expression (mRNA) and TEER data were analyzed for normality of distribution by Kolmogorov-Smirnov test. If distributed normally, means of each group were compared by ANOVA with LSD (homogeneity of variances) or Games-Howell (heterogeneous variances) used as post hoc comparisons. Otherwise, non-parametrical Kruskal Wallis test was carried out. Statistical analyses were performed with SAS (release 9.2; SAS Institute) and SPSS (version 19; SPSS, Inc.). Microbial raw sequencing reads were demultiplexed, quality-filtered and analyzed using QIIME 1.9.1 [34]. Quality-filtered reads were clustered into operational taxonomic units (OTUs) for taxonomy analyses. Taxonomic assignment of representative OTUs was performed using the RDP Classifier [35]. Alignment of sequences was performed using PyNast [36] as default in QIIME pipeline, with an extra filtering step in aligned and taxonomy-assigned OTU table to filter-out sequences that represent less than 0.005% of total OTUs. Downstream analyses were performed at the same depth per sample to standardize for unequal sequencing depth of the samples. Alpha diversity (within group) was assessed using the Shannon index, whose statistical significance was determined with 999 permutations using the non-parametric Monte Carlo permutation test.

Beta diversity (between groups) was assessed calculating weighted UniFrac distances, which were used to conduct principal component analysis. The ANOSIM statistical methods was subsequently applied to evaluate if some variables determined grouping and to which extent. PICRUSt [37] was used to predict the functional profile based on 16S RNA gene sequences. Linear Discriminant Analysis (LDA) Effect Size (LEfSe: https://huttenhower.sph.harvard.edu/galaxy/) was used to compare treatment groups (C+ and OBE) with the negative control (C-) and identify differences in the abundance of predicted functions (α = 0.05 and LDA score > 3.0). Differences were considered significant when *p* < 0.05, whereas when *p* > 0.05 but ≤ 0.10, differences were considered to indicate a trend toward a significant effect.

## Results

### Diet supplementation with OBE attenuates SCI-induced depression of intake and growth

**Fig 1:**
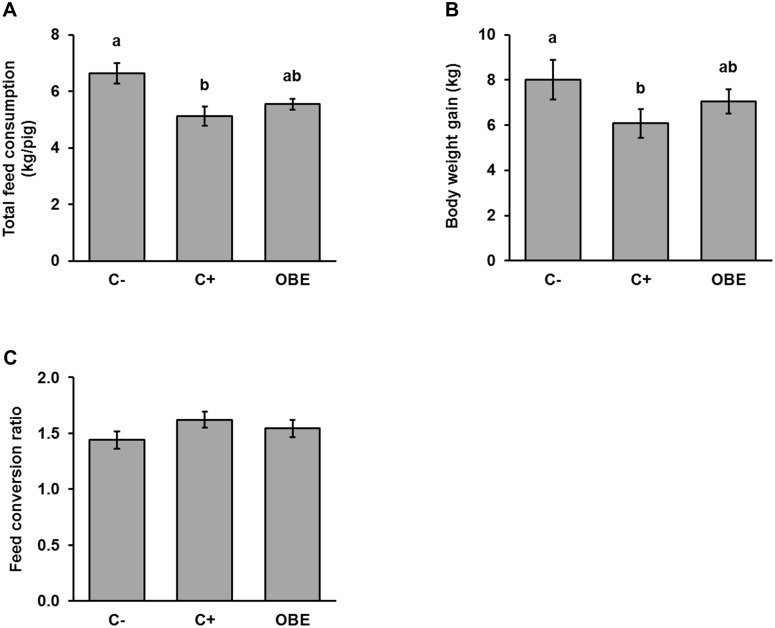
Accumulated feed consumption (kg/pig) and body weight gain (kg) of pigs challenged chronically with LPS and fed an Olive-oil Bioactive Extract (OBE). (A) Feed consumption (kg/pig) of pigs chronically challenged with LPS and fed a commercial pre-starter diet untreated (C-, C+) or supplemented with an olive-oil extract (OBE; 500 mg/kg diet). On d 20, 23, 26 and 29, OBE and positive control (C+) pigs received *E*.*coli*-derived LPS injections at increasing doses (60, 66, 72 and 78 μg/kg). Negative control animals (C-) were injected with saline. (B) Body weight gain (kg) and (C) efficiency of feed conversion (kg of feed consumed/kg of BW gain during the experiment) of the pigs treated as described in A. Bars are least squares means ± SEM (n = 10–11). Different letters indicate significant differences among groups (*p* < 0.05).

During the experimental trial one animal of the OBE-group died (d28). Post-mortem examination did not provide any information on the cause of that mortality. Total feed consumption was significantly decreased from 6.6 ± 0.4 kg/pig to 5.1 ± 0.3 kg/pig by the repeated LPS injections (C- compared to C+, *p* < 0.05). This effect was partly (5.5 ± 0.2 kg/pig; Fig 1A) counteracted by supplementing the diet with OBE. AS projected, LPS injections depressed BW gain significantly by 24%, whereas feeding OBE attenuated such a negative effect (12%; Fig 1B). As a result, exposure to SCI worsened the efficiency of feed conversion by about 13% in sham-treated pigs but only by 7% in OBE-treated animals, although differences among treatments were not significant (Fig 1C).

### Feeding OBE represses the LPS-induced increase in circulating IL1B

**Fig 2:**
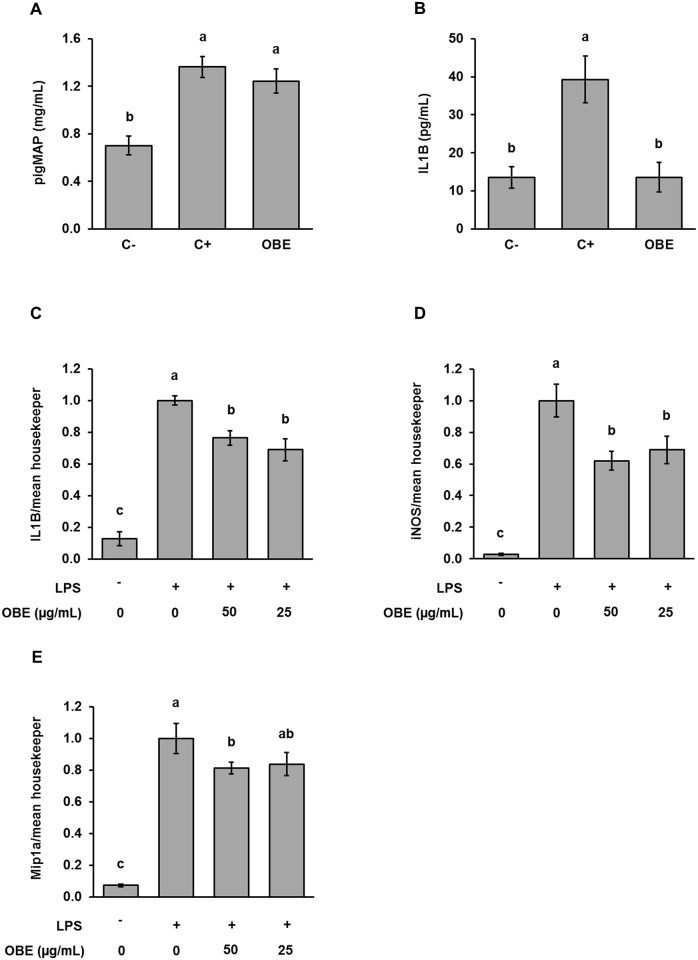
Immune-inflammatory response of pigs and macrophages treated with an Olive-oil Bioactive Extract (OBE) and challenged with LPS. (A, B) Concentration of pigMAP and ILB1 in peripheral circulation of pigs. Piglets were fed a commercial diet untreated (C-, C+) or supplemented with an olive-oil extract (OBE; 500 mg/kg diet). On d 20, 23, 26 and 29, OBE and positive control (C+) pigs received *E*.*coli*-derived LPS injections at increasing doses (60, 66, 72 and 78 μg/kg). Negative control animals (C-) were injected with saline. Plasma samples were collected 3 h after final LPS administration and pigMAP and cytokine levels were determined by sandwich ELISA. Bars are least squares means ± SEM (pigMAP, n = 10–11, IL1B, n = 3–6). (C, D, E) Expression of IL1B, iNOS and Mip1a genes in RAW 264.7 macrophages. Cells were treated with OBE (50 and 25 μg/mL) and DMSO (0.1% v/v) for 24 h and subsequently challenged with LPS (10 ng/mL) for 6 h. The concentration of mRNA was measured *via* qRT-PCR. Bars represent means ± SEM of 3 independent experiments performed in duplicate. Values are normalized to the sham-treated control. Different letters indicate significant differences among groups (*p* < 0.05).

Compared to untreated pigs (C-), the repeated administration of LPS to C+ animals raised significantly the circulating concentration of pigMAP by almost 2 fold (0.70 vs. 1.36 mg/mL, Fig 2A), confirming that the experimental model successfully caused a systemic and chronic activation of the immune system. This response was not affected by the feeding of OBE (Fig 2A). In some (n = 4), but not all animals LPS injection resulted in an increase of IL1B concentration in plasma. Supplementing the diet with OBE partly (n = 5) abrogated the increase in plasma IL1B otherwise triggered by the chronic challenge with LPS (C+ = 39 vs. C- = 13 pg/mL; *p* < 0.001; Fig 2B). To confirm the *in vivo* anti-inflammatory effect of OBE we challenged macrophages with LPS *in vitro*, thereby inducing the expression of the pro-inflammatory markers IL1B (Fig 2C), nitric oxide synthase 2 (iNOS; Fig 2D) and macrophage inflammatory protein 1 alpha (Mip1a; Fig 2E). Pre-exposing macrophages to OBE significantly attenuated LPS-induced inflammatory response by 20 to 40% irrespective of the concentration of OBE (Fig 2C, 2D and 2E).

### Both chronic systemic LPS challenge and OBE do not affect intestinal inflammation

**Fig 3:**
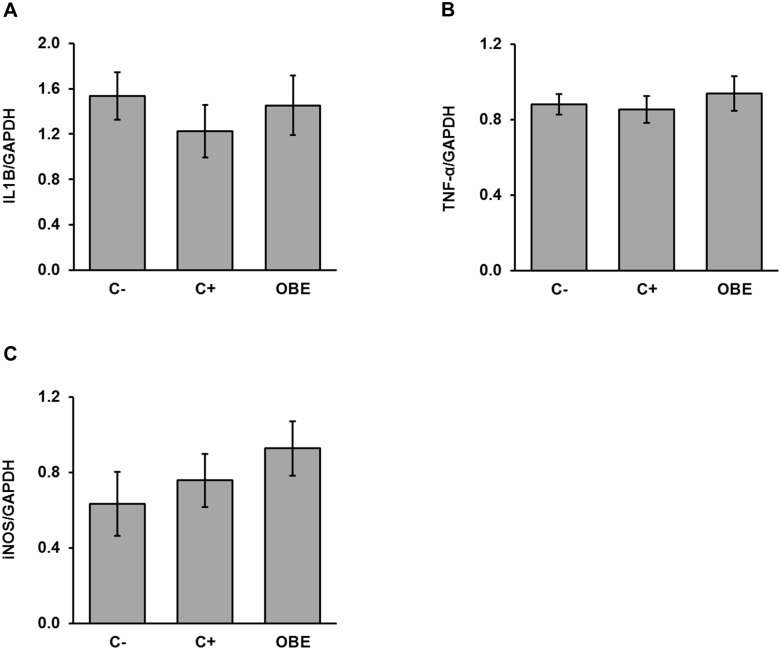
Relative concentrations of IL1B (A), TNF-α (B), and iNOS (C) mRNA in ileal mucosa of pigs challenged chronically with LPS and fed an Olive-oil Bioactive Extract (OBE). Piglets were fed a commercial diet either untreated (C-, C+) or supplemented with an olive-oil extract (OBE; 500 mg/kg diet). On d 20, 23, 26 and 29, OBE and positive control (C+) pigs received *E*.*coli*-derived LPS injections at increasing doses (60, 66, 72 and 78 μg/kg). Negative control animals (C-) were injected with saline. Mucosa samples were collected 3 h after final LPS administration and mRNA levels of the abovementioned markers were measured *via* qRT-PCR. Bars are means ± SEM (n = 9–10).

In order to elucidate if the repeated i.p. injections of LPS and the feeding of OBE within such an experimental setting alter intestinal inflammation, the mRNA abundance of selected pro-inflammatory markers were measured in ileal mucosa. Transcript levels of IL1B, TNF-α and iNOS did not reveal any significant change in ileal inflammation in response to the experimental model or dietary treatment (Fig 3).

### OBE improves intestinal integrity in pigs chronically challenged with LPS

**Fig 4:**
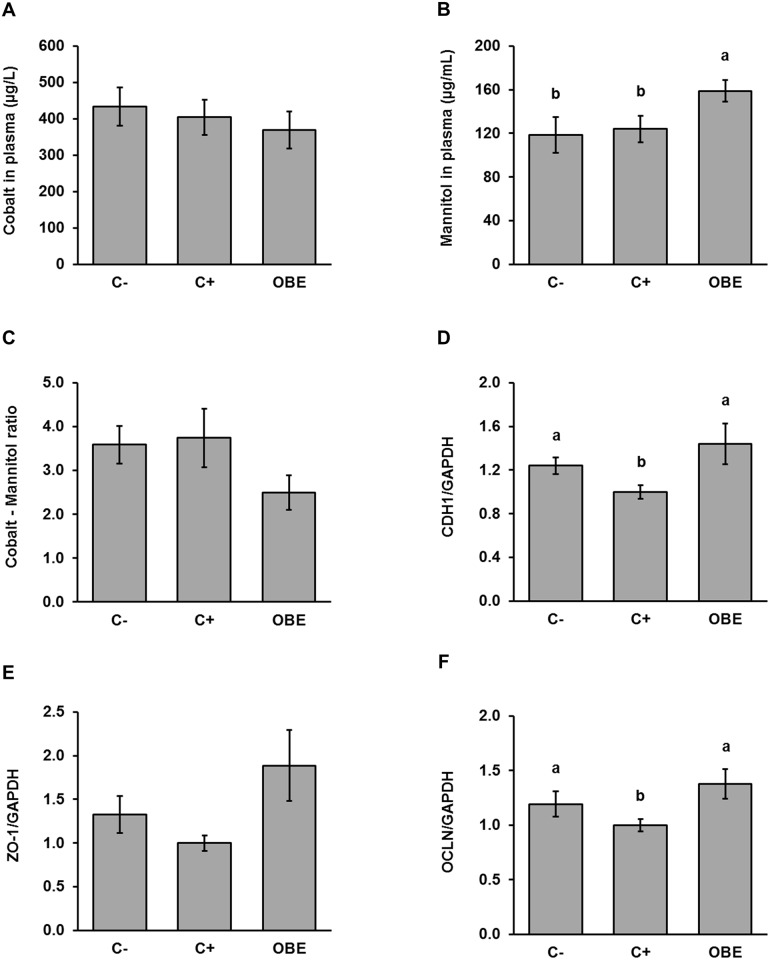
Concentrations of permeability markers (A-C) in plasma and relative concentrations of CDH1; (D), OCLN (E), and ZO-1 (F) in the ileal mucosa of pigs challenged chronically with LPS and fed an Olive-oil Bioactive Extract (OBE). Piglets were fed a standard diet either untreated (C-, C+) or supplemented with an olive-oil extract (OBE; 500 mg/kg diet). On d 20, 23, 26 and 29, OBE and positive control (C+) pigs received *E*.*coli*-derived LPS injections at increasing doses (60, 66, 72 and 78 μg/kg). Negative control animals (C-) were injected with saline. Plasma samples were collected *via* jugular venipuncture 1 h after intragastric infusion of the marker solution containing 0.15 g mannitol/kg BW and 0.1 g cobalt-EDTA/kg BW. Mucosa samples were collected 3 h after final LPS administration and mRNA levels of junctional proteins were measured *via* qRT-PCR. Bars are least squares means ± SEM (n = 9–11). Different letters indicate significant differences among groups (*p* < 0.05).

**Fig 5:**
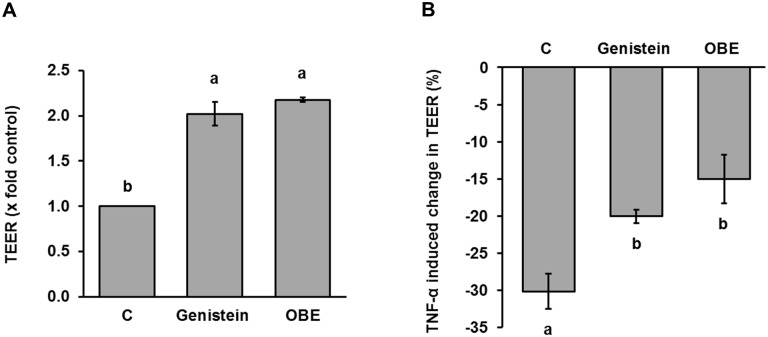
Effect of an olive-oil bioactive extract on basal TEER (A) and TNF-α induced decrease in TEER (B) *in vitro*. Caco-2/TC-7 cells were allowed to differentiate on permeable filters in the presence of an olive-oil bioactive extract (OBE; 100 μg/mL), genistein (50 μmol/L; positive control), and DMSO (0.1% v/v; vehicle control [C]) for 5 d. (A) Barrier integrity was assessed by measuring the TEER over time. To investigate potential protective effects of OBE on Caco-2/TC-7 barrier integrity, TNF-α (100 ng/mL) was added to the basolateral side for 24 h on d 5 to induce monolayer disruption. (B) TEER was measured before and after TNF-α administration and values were normalized to the untreated control. Bars represent means of 3 individual experiments ± SEM. Different letters indicate significant differences among treatments (*p* < 0.05).

Intestinal permeability was investigated by infusing pigs intragastrically with a marker solution containing mannitol and cobalt-EDTA. Plasma recovery of these permeability markers was not significantly affected by the chronic LPS challenge, indicating that the experimental model did not impair the paracellular and transcellular transport routes (Fig 4A and 4B). Interestingly, the highest (*p* < 0.05) concentration of mannitol was found in animals receiving the OBE treatment (Fig 4C). As a result, feeding OBE tended (*p* = 0.12) to reduce the ratio between markers compared to both C- and C+ (Fig 4C). To determine if the impact of OBE on mannitol permeation was associated with structural alterations of the intestinal mucosa, gene expression of tight-junctional proteins was measured in the ileum. The relative concentration of CDH1 mRNA (Fig 4D), but not that of ZO-1 (Fig 4E) and OCLN (Fig 4F), was significantly decreased by 25% in C+ animals compared to untreated controls. Notably, the feeding of OBE rescued or even enhanced the mRNA level of CDH1, OCLN and ZO-1 in ileal mucosa in comparison to sham-treated animals (Fig 4D–4F). Furthermore, OBE-fed pigs also tended to have higher transcript levels of OCLN and ZO-1 than C- animals (Fig 4E and 4F). In stark agreement with *in vivo* results, treatment of human Caco-2/TC-7 monolayers grown on semipermeable membranes with OBE resulted in a significant increase in TEER values comparable to genistein which was used as the positive control (Fig 5A). Simultaneous administration of TNF-α induced a significant loss of barrier integrity as indicated by a drop in TEER of 30%. OBE significantly counteracted TNF-α mediated barrier disruption (Fig 5B) comparable to the positive control genistein. Taken together, these results indicate that the integrity of the intestinal mucosa of pigs and Caco-2/TC-7 cells was improved by OBE.

### Gut microbial composition and functions are unaffected by OBE and chronic LPS challenge

**Fig 6:**
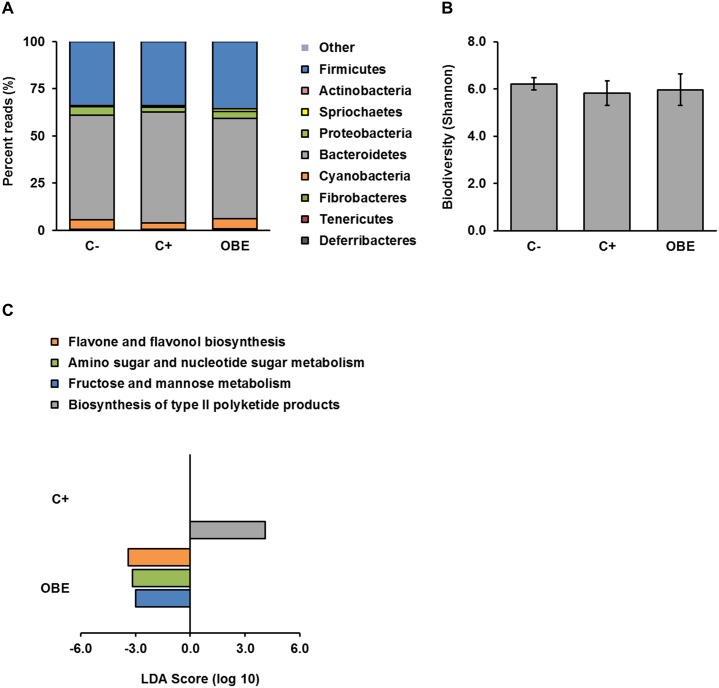
Gut microbial composition and predicted functions in pigs chronically challenged with LPS and fed an Olive-oil Bioactive Extract (OBE). (A) Percent reads at the phylum level in colonic contents resulting from the 16S rRNA gene sequencing analysis. (B) Diversity of colonic microbiota within groups of pigs (alpha diversity; C- vs. C+ *p* < 0.12, C- vs. OBE *p* < 1.0, C+ vs. OBE *p* < 1.0). (C) Linear discriminant analysis (LDA) scores for microbial functions predicted by PICRUSt (α = 0.05, LDA score > 3.0). Piglets were fed a standard diet either untreated (C-, C+) or supplemented with an olive-oil extract (OBE; 500 mg/kg diet). On d 20, 23, 26 and 29, OBE and positive control (C+) pigs received *E*.*coli*-derived LPS injections at increasing doses (60, 66, 72 and 78 μg/kg). Negative control animals (C-) were injected with saline. Samples of colonic content were collected 3 h after final LPS administration and analyzed *via* massive sequencing of the V1-V2 hypervariable regions of the 16S rRNA gene (n = 9–11).

Provided that some bioactive compounds in olive oil have antimicrobial actions [11] and that their health-promoting effects may require interaction with the gut microbiota [21], we aimed to elucidate if the mode of action of OBE involves changes in the colonic microbial ecology of LPS-challenged pigs by using massive sequencing. At the phylum level, all treatments groups presented normal [38] and similar structures of the gut microbiome (Fig 6A). In addition, metrics of biodiversity within (Shannon index, Fig 6B) and among groups (weighted UniFrac distances, ANOSIM *p* < 0.743) of animals were not significantly different. Furthermore, both OBE and the repeated administration of LPS (C+) elicited minor changes in microbial functions as predicted by PICRUSt (Fig 6C). It appears therefore that in our study gut microbial composition and metabolism remained rather constant among groups of pigs.

## Discussion

Our data indicate that dietary supplementation with OBE can alleviate detrimental effects of SCI by modulating the immune-inflammatory response of pigs and thereby inducing persistent effects on animal health and performance.

According to recent literature, repeated intraperitoneal injections of bacterial LPS at low doses represent a suitable approach to create a phenotype exhibiting typical characteristics of SCI in weaned piglets [24]. More precisely, these characteristics include partial suppression of feed intake and BW gain as well as elevation of inflammatory markers in peripheral circulation, including cytokines and APP [23; 39]. In contrast to models of acute inflammation, in which LPS is usually administered in a single dose of about 120–180 mg/kg BW, reduction of feed intake is less pronounced but persists over the challenge period [40; 41]. In line with these reports, we also observed that the intraperitoneal challenge with low doses of LPS (60 to 78 mg/kg BW) significantly suppressed feed consumption and BW gain in C+ pigs compared to untreated controls (C-). In addition, this response was associated with enhanced levels of pigMAP and ILB1 in systemic circulation. These observations confirm that the experimental model used in our study successfully induce SCI as previously reported. Under this experimental setting, supplementation of the diet with OBE attenuated the negative impact of SCI on animal performance (i.e., improved feed ingestion and growth) and repressed the LPS-induced increase in circulating IL1B without affecting pigMAP. Interestingly, these effects were paralleled by an improvement in intestinal integrity otherwise compromised by SCI. Furthermore, from a mechanistic standpoint the actions of OBE were apparently independent of alterations in gut microbial ecology.

Under inflammatory conditions acute immune response is followed by the synthesis and release of different APP, including C-reactive protein, haptoglobulin, alkaline phosphatase and pigMAP [42]. Although their exact physiological actions have not been entirely elucidated, APP seem to control inflammatory response [43]. As an APP specific to infections [44], pigMAP was found to be significantly raised in our sham-treated piglets as compared to controls, providing further evidence that the experimental model induced SCI. The finding that OBE did not affect such a response indicates that the synthesis and secretion of APP is not a mechanistic target of its anti-inflammatory action. The effects of IL1B on feeding behavior are well-known and diverse, including decreased gastric emptying and motility as well as direct actions on the central nervous system [45; 46]. Considering that IL1B serves as one of the main suppressors of feed intake in response to inflammation [47; 48; 49], in the study reported herein it seems plausible to associate decreased animal performance with the partly increased plasma concentration of IL1B. Remarkably, the increase in IL1B and the concurrent reduction in feed ingestion were not observed in LPS-challenged animals receiving the OBE treatment, suggesting that the growth permitting effect of OBE under SCI involves modulation of the inflammatory response.

In support of these findings, we also found compelling evidence of anti-inflammatory effects of OBE in cultured cells. In murine RAW 264.7 macrophages LPS is known to trigger an inflammatory response *via* activation of the NF-kB pathway [50; 51]. Accordingly, we observed a significant increase in the mRNA level of the NF-kB target genes IL1B and iNOS 6 h post challenge with LPS (10 ng/mL). Treating macrophages with OBE before the stimulation with LPS partly counteracted such responses by about 30%. These results are similar to findings from recent studies demonstrating anti-inflammatory properties of polyphenols and other plant bioactives in cell cultures [52; 53] and farm animals [54]. Furthermore, our *in vivo* and *in vitro* results indicate that inhibition of IL1B production is a key component of the OBE anti-inflammatory action. Interestingly, the anti-IL1B activity of OBE seems to be higher *in vivo* than *in vitro*. Considering that OBE could not have undergone major metabolism *in vitro*, it seems reasonable to expect that extensive transformations of OBE *in vivo* might have occurred thereby enhancing its bioactivity. Additionally, we found that the gene expression of pro-inflammatory cytokines, including IL1B, in ileal mucosa remained constant among experimental groups. Therefore, data indicate that both the experimental setting and dietary treatment did not modulate the immune-inflammatory axis locally within the intestine. The intestinal absorption of polyphenols is known to be rather low [55; 56] which may question the impact of dietary polyphenols on systemic immune response. However, since secondary plant metabolites, including those of *Olea europaea* are intensively metabolized before entering systemic circulation glucuronide and sulfate conjugates may significantly contribute to potential *in vivo* effects [57; 58].

Although intestinal inflammation was not apparent in this study, several pieces of evidence demonstrate that OBE improved the integrity of the intestinal mucosa of pigs challenged with SCI. From a functional view point, supplementing the diet with OBE enhanced permeation of the inert probe mannitol while tended to reduce the ratio between permeability markers compared to both control groups. Provided that the extent of permeation of these inert markers *in vivo* permits to discriminate between the paracellular (Co-EDTA) and transcellular (mannitol) pathways of transepithelial transport and that their ratio gives information about the integrity of the gastrointestinal epithelium [59; 60], these findings denote that OBE improved the functional capacity for molecular sieving of the intestinal mucosa of SCI-affected animals. This ability was later confirmed *in vitro* by showing that OBE partly prevented disruption of the transjunctional flux of ions (TEER) in TNF-α-challenged Caco-2/TC-7 cells, a model that resembles impairment of intestinal barrier function associated with chronic inflammation [61; 62; 63]. The concentration of OBE, as used within this study, is comparable to the ones used for other extracts in Caco-2-cell culture studies [64]. Interestingly, the finding that the magnitude of the improvement on gut mucosal integrity even exceeded the negative control group suggests that OBE may exert such an action in the absence of SCI. Furthermore, the protective effect of OBE even exceeded that of genistein, an isoflavonoid known for its barrier strengthening action in epithelial cell lines [65; 66] and used as a positive control in the present study. Similar findings have been reported by Piegholdt and coworkers [67], who showed a significant increase in TEER through treatment of cell cultures with hydroxytyrosol, a bioactive phenolic molecule found in *Olea europaea*. In relation to structural aspects of the intestinal mucosa, administration of OBE increased gene expression of proteins (CDH1, ZO-1, OCLN) that form apical junctional complexes [68] in the ileum of SCI-challenged pigs. Pro-inflammatory cytokines compromise intestinal barrier and gut health partly by regulating the expression of such junctional proteins [68; 69; 70], suggesting that reduced IL1B in peripheral circulation might be a mechanistic component of the enteroprotective action of OBE. It is important to note, however, that IL1B [68; 71] and other pro-inflammatory cytokines [72; 73; 74] disrupt barrier integrity by enhancing the expression and activity of myosin light chain kinase and thereby inducing contraction of the cytoskeleton. In addition, olive-derived polyphenols and triterpenoids may mediate cytoprotective actions via inhibition of NF-kB and/or activation of the Nrf2 pathways [75; 76; 77]. Based on its chemical composition, it is likely that the described impact of OBE on gut mucosal integrity might have involved modulation of such signaling cascades.

In the past decade the intestinal microbiome has gained massive scientific attention because of the complex crosstalk between gut microbiota and host metabolism [78; 79]. Recent work has shown that modifications of the abundance and diversity of certain microbial taxa is associated with increased intestinal permeability and thus contribute to SCI through elevated migration of bacterial antigens into systemic circulation [80; 81; 82]. A second line of evidence indicates that plant polyphenols, including those from *Olea europaea*, require interaction with the intestinal microbiota to exert some, if not all, of their health-promoting effects which expand beyond the proper functioning of the intestinal barrier [21]. Furthermore, a number of bioactive compounds present in different parts of the olive tree possess inhibitory activity against some gut-resident bacteria [11].

In view of this knowledge, we hypothesized that the protective action of OBE against SCI may involve changes in the structure and/or function of the gut microbiome. We observed, however, that the composition, diversity, and predicted functions of colonic microbiota were similar among treatment groups, suggesting that the anti-SCI effects of OBE are not mediated through changes in gut microbial ecology. Nonetheless, the interplay between plant bioactives and gut microbes is bidirectional [21]; therefore, the implication of potential microbial transformations of bioactive compounds in OBE cannot be ruled out.

We suggest that our pig data may have also relevance to human health and disease. There is a high degree of similarity in terms of anatomy, physiology, immune function and gut microbiota between pigs and humans [83]. Furthermore pigs exhibit similar syndromes to humans including intestinal inflammation and diarrhea [84].

## Conclusion

**Fig 7:**
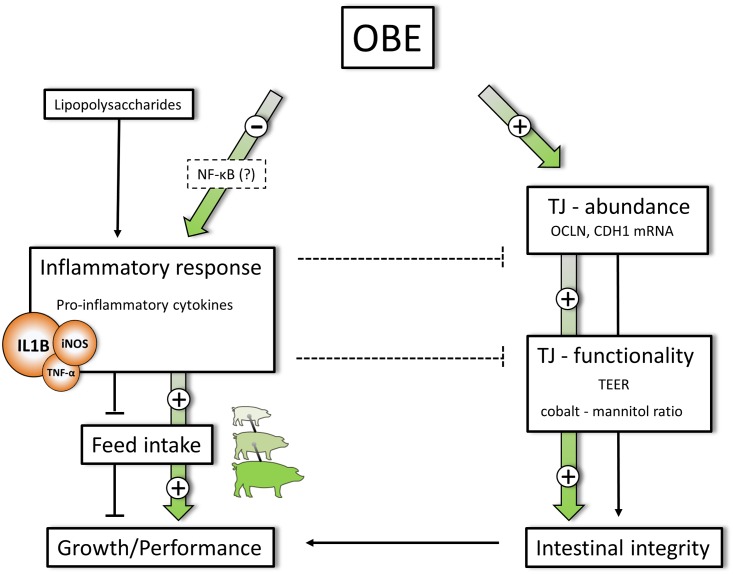
Supposed metabolic targets involved in OBE mediated effects. Repeated LPS injection stimulates the systemic secretion of pro-inflammatory IL1B and simultaneously suppresses feed intake and growth in challenged animals. OBE is capable of counteracting LPS stimulated IL1B secretion most likely through interaction with NF-κB signal cascade. OBE treatment further increases the concentration of junctional mRNA (OCLN, CDH1, ZO-1) and promotes TJ-functionality as indicated by decreased ion flux (TEER) and improved cobalt to mannitol ratio. Increased gene expression of junctional proteins as well as an improved TJ-functionality can be linked to enhanced gut integrity, further supporting animal growth and performance. Particular importance is devoted to the finding that the growth promoting effect of OBE is mediated independent of changes in gut microbial composition and diversity. Plain connections represent observed (solid) and supposed (interrupted) metabolic effects of chronic LPS challenge. Colour-filled arrows indicate effects of OBE treatment.

Overall, data reported herein demonstrate that olive-derived bioactive compounds have growth-permitting action in pigs challenged experimentally with SCI. The underlying mode of action apparently includes anti-inflammatory effects, in particular inhibition of IL1B production and protection of intestinal integrity unrelated to alterations in gut microbial ecology (Fig 7). Taken together, supplementing the diet of pigs with OBE might represent a promising strategy to counteract SCI-related disorders in commercial settings of pig production.

## Supporting information

S1 Fig Cytotoxicity of the Olive-oil Bioactive Extract (OBE) in Caco-2/TC-7 (A) and RAW 264.7 (B) cells. Cytotoxicity of OBE as assessed by neutral red assay and expressed as remaining cell viability after treatment. Cells were incubated with DMSO (0.1% v/v, C-), ethanol (10%, C+) or increasing concentrations of OBE. Bars represent means ± SEM of 2 independent experiments performed in triplicate. Different letters indicate significant differences among treatments (*p* < 0.05).
(TIF)

## Abbreviations

APP: acute-phase protein
BW: body weight
C-: negative control
C+: positive control
CDH1: E-cadherin
ELISA: Enzyme-linked Immunosorbent Assay
GAPDH: glyceraldehyde-3-phosphate dehydrogenase
HPLC: high-performance liquid chromatography
i.m.: intramuscular
i.p.: intraperitoneal
IL1B: interleukin 1 beta
IL8: interleukin-8
iNOS: nitric oxide synthase 2
LPS: lipopolysaccharide
Mip1a: macrophage inflammatory protein 1 alpha
OBE: olive-oil bioactive extract
OCLN: occludin
qRT-PCR: quantitative RT-PCR
TEER: transepithelial electrical resistance
TJ: tight junction
TMB: 3, 3’, 5, 5’–tetramethylbenzidin
TNF-α: tumor necrosis factor alpha
ZO-1: zonula occludens 1

## References

[1] Medzhitov R. Origin and physiological roles of inflammation. Nature. 2008; 454: 428–435. 10.1038/nature07201 18650913

[2] Jiminez JA, Uwiera TC, Douglas Inglis G, Uwiera RRE. Animal models to study acute and chronic intestinal inflammation in mammals. Gut Pathog. 2015; 7: 29 10.1186/s13099-015-0076-y 26561503 PMC4641401

[3] Murakami M, Hirano T. The molecular mechanisms of chronic inflammation development. Front Immunol. 2012; 3: 323 10.3389/fimmu.2012.00323 23162547 PMC3498841

[4] Ashley NT, Weil ZM, Nelson RJ. Inflammation. Mechanisms, Costs, and Natural Variation. Annu. Rev. Ecol. Evol. Syst. 2012; 43: 385–406.

[5] Dinarello CA. Historical insights into cytokines. Eur J Immunol. 2007; 37 Suppl 1: S34–45. 17972343 10.1002/eji.200737772 PMC3140102

[6] Mantovani G, Anker SD, Inui A, Morley JE, Fanelli FR, Scevola D, et al Cachexia and Wasting: A Modern Approach. Milano: Springer Milan; 2006.

[7] Papadakis KA, Targan SR. Role of cytokines in the pathogenesis of inflammatory bowel disease. Annu Rev Med. 2000; 51: 289–298. 10.1146/annurev.med.51.1.289 10774465

[8] Feghali CA, Wright TM. Cytokines in acute and chronic inflammation. Front Biosci. 1997; 2: d12–26. 9159205 10.2741/a171

[9] Hanada T, Yoshimura A. Regulation of cytokine signaling and inflammation. Cytokine & Growth Factor Reviews. 2002; 13: 413–421. 12220554 10.1016/s1359-6101(02)00026-6

[10] Rakhshandeh A, Htoo JK, de Lange C. Immune system stimulation of growing pigs does not alter apparent ileal amino acid digestibility but reduces the ratio between whole body nitrogen and sulfur retention. Livestock Science. 2010; 134: 21–23.

[11] Ghanbari R, Anwar F, Alkharfy KM, Gilani A-H, Saari N. Valuable nutrients and functional bioactives in different parts of olive (Olea europaea L.)-a review. Int J Mol Sci. 2012; 13: 3291–3340. 10.3390/ijms13033291 22489153 PMC3317714

[12] Bayram B, Ozcelik B, Schultheiss G, Frank J, Rimbach G. A validated method for the determination of selected phenolics in olive oil using high-performance liquid chromatography with coulometric electrochemical detection and a fused-core column. Food Chemistry. 2013; 138: 1663–1669. 10.1016/j.foodchem.2012.11.122 23411296

[13] Bayram B, Esatbeyoglu T, Schulze N, Ozcelik B, Frank J, Rimbach G. Comprehensive analysis of polyphenols in 55 extra virgin olive oils by HPLC-ECD and their correlation with antioxidant activities. Plant Foods Hum Nutr. 2012; 67: 326–336. 10.1007/s11130-012-0315-z 23070730

[14] Piroddi M, Albini A, Fabiani R, Giovannelli L, Luceri C, Natella F, et al Nutrigenomics of extra-virgin olive oil: A review. Biofactors. 2016. 10.1002/biof.1318 27580701

[15] Visioli F, Galli C. Biological properties of olive oil phytochemicals. Crit Rev Food Sci Nutr. 2002; 42: 209–221. 10.1080/10408690290825529 12058980

[16] Sanchez-Quesada C, Lopez-Biedma A, Warleta F, Campos M, Beltran G, Gaforio JJ. Bioactive properties of the main triterpenes found in olives, virgin olive oil, and leaves of Olea europaea. J Agric Food Chem. 2013; 61: 12173–12182. 10.1021/jf403154e 24279741

[17] Caramia G, Gori A, Valli E, Cerretani L. Virgin olive oil in preventive medicine. From legend to epigenetics. Eur. J. Lipid Sci. Technol. 2012; 114: 375–388.

[18] El SN, Karakaya S. Olive tree (Olea europaea) leaves: potential beneficial effects on human health. Nutr Rev. 2009; 67: 632–638. 10.1111/j.1753-4887.2009.00248.x 19906250

[19] Pereira AP, Ferreira IC, Marcelino F, Valentão P, Andrade PB, Seabra R, et al Phenolic Compounds and Antimicrobial Activity of Olive (Olea europaea L. Cv. Cobrançosa) Leaves. Molecules. 2007; 12: 1153–1162. 17873849 10.3390/12051153 PMC6149345

[20] Benavente-García O, Castillo J, Lorente J, Ortuño A, Del Rio J. Antioxidant activity of phenolics extracted from Olea europaea L. leaves. Food Chemistry. 2000; 68: 457–462.

[21] Cardona F, Andres-Lacueva C, Tulipani S, Tinahones FJ, Queipo-Ortuno MI. Benefits of polyphenols on gut microbiota and implications in human health. J Nutr Biochem. 2013; 24: 1415–1422. 10.1016/j.jnutbio.2013.05.001 23849454

[22] Morton DB, Griffiths PH. Guidelines on the recognition of pain, distress and discomfort in experimental animals and an hypothesis for assessment. Vet Rec. 1985; 116: 431–436. 3923690 10.1136/vr.116.16.431

[23] Rakhshandeh A, de Lange C F M. Evaluation of chronic immune system stimulation models in growing pigs. Animal. 2012; 6: 305–310. 10.1017/S1751731111001522 22436189

[24] Sánchez P, Dobarganes MA, Ruiz-Méndez MV. Edible olive pomace oil concentrated in triterpenic acids, procedure of physical refining utilised for obtainment thereof and recovery of functional components present in the crude oil. US Patent No.: US8361518B2

[25] Romero C, García A, Medina E, Ruíz-Méndez MV, Castro Ad, Brenes M. Triterpenic acids in table olives. Food Chemistry. 2010; 118: 670–674.

[26] Mulinacci N, Romani A, Galardi C, Pinelli P, Giaccherini C, Vincieri FF. Polyphenolic Content in Olive Oil Waste Waters and Related Olive Samples. J. Agric. Food Chem. 2001; 49: 3509–3514. 11513620 10.1021/jf000972q

[27] Uden P, Colucci PE, van Soest PJ. Investigation of chromium, cerium and cobalt as markers in digesta. Rate of passage studies. J Sci Food Agric. 1980; 31: 625–632. 6779056 10.1002/jsfa.2740310702

[28] Mereu A, Tedo G, Moeser AJ, Rimbach G, Ipharraguerre IR. Cromolyn-mediated improvement of intestinal barrier function is associated with enhanced piglet performance after weaning. BMC Vet Res. 2015; 11: 274 10.1186/s12917-015-0588-1 26510713 PMC4624645

[29] Chantret I, Rodolosse A, Barbat A, Dussaulx E, Brot-Laroche E, Zweibaum A, et al Differential expression of sucrase-isomaltase in clones isolated from early and late passages of the cell line Caco-2: evidence for glucose-dependent negative regulation. J Cell Sci. 1994; 107 (Pt 1): 213–225. 8175910 10.1242/jcs.107.1.213

[30] Ralph P, Nakoinz I. Antibody-dependent killing of erythrocyte and tumor targets by macrophage-related cell lines: enhancement by PPD and LPS. J Immunol. 1977; 119: 950–954. 894031

[31] Borenfreund E, Shopsis C. Toxicity monitored with a correlated set of cell-culture assays. Xenobiotica. 1985; 15: 705–711. 10.3109/00498258509047431 4072257

[32] Hidalgo IJ, Raub TJ, Borchardt RT. Characterization of the human colon carcinoma cell line (Caco-2) as a model system for intestinal epithelial permeability. Gastroenterology. 1989; 96: 736–749. 2914637

[33] Srinivasan B, Kolli AR, Esch MB, Abaci HE, Shuler ML, Hickman JJ. TEER measurement techniques for in vitro barrier model systems. J Lab Autom. 2015; 20: 107–126. 10.1177/2211068214561025 25586998 PMC4652793

[34] Caporaso JG, Kuczynski J, Stombaugh J, Bittinger K, Bushman FD, Costello EK, et al QIIME allows analysis of high-throughput community sequencing data. Nat Methods. 2010; 7: 335–336. 10.1038/nmeth.f.303 20383131 PMC3156573

[35] Wang Q, Garrity GM, Tiedje JM, Cole JR. Naive Bayesian classifier for rapid assignment of rRNA sequences into the new bacterial taxonomy. Appl Environ Microbiol. 2007; 73: 5261–5267. 10.1128/AEM.00062-07 17586664 PMC1950982

[36] Caporaso JG, Bittinger K, Bushman FD, DeSantis TZ, Andersen GL, Knight R. PyNAST: a flexible tool for aligning sequences to a template alignment. Bioinformatics. 2010; 26: 266–267. 10.1093/bioinformatics/btp636 19914921 PMC2804299

[37] Langille MGI, Zaneveld J, Caporaso JG, McDonald D, Knights D, Reyes JA, et al Predictive functional profiling of microbial communities using 16S rRNA marker gene sequences. Nat Biotechnol. 2013; 31: 814–821. 10.1038/nbt.2676 23975157 PMC3819121

[38] Kim HB, Isaacson RE. The pig gut microbial diversity: Understanding the pig gut microbial ecology through the next generation high throughput sequencing. Vet Microbiol. 2015; 177: 242–251. 10.1016/j.vetmic.2015.03.014 25843944

[39] Pastorelli H, van Milgen J, Lovatto P, Montagne L. Meta-analysis of feed intake and growth responses of growing pigs after a sanitary challenge. Animal. 2012; 6: 952–961. 10.1017/S175173111100228X 22558966

[40] Cray C, Zaias J, Altman NH. Acute phase response in animals: a review. Comp Med. 2009; 59: 517–526. 20034426 PMC2798837

[41] van Heugten E, Spears JW, Coffey MT. The effect of dietary protein on performance and immune response in weanling pigs subjected to an inflammatory challenge. J Anim Sci. 1994; 72: 2661–2669. 7883625 10.2527/1994.72102661x

[42] Wyns H, Plessers E, de Backer P, Meyer E, Croubels S. In vivo porcine lipopolysaccharide inflammation models to study immunomodulation of drugs. Vet Immunol Immunopathol. 2015; 166: 58–69. 10.1016/j.vetimm.2015.06.001 26099806

[43] Jain S, Gautam V, Naseem S. Acute-phase proteins: As diagnostic tool. J Pharm Bioallied Sci. 2011; 3: 118–127. 10.4103/0975-7406.76489 21430962 PMC3053509

[44] Pomorska-Mol M, Kwit K, Pejsak Z, Markowska-Daniel I. Analysis of the acute-phase protein response in pigs to clinical and subclinical infection with H3N2 swine influenza virus. Influenza Other Respir Viruses. 2014; 8: 228–234. 10.1111/irv.12186 24734294 PMC4186471

[45] Suto G, Kiraly A, Tache Y. Interleukin 1 beta inhibits gastric emptying in rats: mediation through prostaglandin and corticotropin-releasing factor. Gastroenterology. 1994; 106: 1568–1575. 8194703 10.1016/0016-5085(94)90412-x

[46] Plata-Salaman CR, Oomura Y, Kai Y. Tumor necrosis factor and interleukin-1 beta: suppression of food intake by direct action in the central nervous system. Brain Res. 1988; 448: 106–114. 3260533 10.1016/0006-8993(88)91106-7

[47] Plata-Salaman CR. Immunoregulators in the nervous system. Neurosci Biobehav Rev. 1991; 15: 185–215. 1852312 10.1016/s0149-7634(05)80001-6

[48] Swiergiel AH, Dunn AJ. The roles of IL-1, IL-6, and TNFalpha in the feeding responses to endotoxin and influenza virus infection in mice. Brain Behav Immun. 1999; 13: 252–265. 10.1006/brbi.1999.0565 10469526

[49] Bret-Dibat JL, Bluthe RM, Kent S, Kelley KW, Dantzer R. Lipopolysaccharide and interleukin-1 depress food-motivated behavior in mice by a vagal-mediated mechanism. Brain Behav Immun. 1995; 9: 242–246. 8590821 10.1006/brbi.1995.1023

[50] Jofre-Monseny L, Loboda A, Wagner AE, Huebbe P, Boesch-Saadatmandi C, Jozkowicz A, et al Effects of apoE genotype on macrophage inflammation and heme oxygenase-1 expression. Biochem Biophys Res Commun. 2007; 357: 319–324. 10.1016/j.bbrc.2007.03.150 17416347 PMC2096715

[51] Caamano J, Hunter CA. NF-kappaB family of transcription factors: central regulators of innate and adaptive immune functions. Clin Microbiol Rev. 2002; 15: 414–429. 10.1128/CMR.15.3.414-429.2002 12097249 PMC118079

[52] Hwang J-H, Lim S-B. Antioxidant and Anti-inflammatory Activities of Broccoli Florets in LPS-stimulated RAW 264.7 Cells. Prev Nutr Food Sci. 2014; 19: 89–97. 10.3746/pnf.2014.19.2.089 25054107 PMC4103733

[53] Chen Y, Lin Y, Li Y, Li C. Total flavonoids of Hedyotis diffusa Willd inhibit inflammatory responses in LPS-activated macrophages via suppression of the NF-kappaB and MAPK signaling pathways. Exp Ther Med. 2016; 11: 1116–1122. 10.3892/etm.2015.2963 26998046 PMC4774565

[54] Gessner DK, Ringseis R, Eder K. Potential of plant polyphenols to combat oxidative stress and inflammatory processes in farm animals. J Anim Physiol Anim Nutr (Berl). 2016. 10.1111/jpn.12579 27456323

[55] Scalbert A, Morand C, Manach C, Rémésy C. Absorption and metabolism of polyphenols in the gut and impact on health. Biomedicine & Pharmacotherapy. 2002; 56: 276–282. 12224598 10.1016/s0753-3322(02)00205-6

[56] Marin L, Miguelez EM, Villar CJ, Lombo F. Bioavailability of dietary polyphenols and gut microbiota metabolism: antimicrobial properties. Biomed Res Int. 2015; 2015: 905215 10.1155/2015/905215 25802870 PMC4352739

[57] Pastor A, Rodriguez-Morato J, Olesti E, Pujadas M, Perez-Mana C, Khymenets O, et al Analysis of free hydroxytyrosol in human plasma following the administration of olive oil. J Chromatogr A. 2016; 1437: 183–190. 10.1016/j.chroma.2016.02.016 26877176

[58] Catalan U, Lopez de Las Hazas Maria-Carmen, Rubio L, Fernandez-Castillejo S, Pedret A, de La Torre R, et al Protective effect of hydroxytyrosol and its predominant plasmatic human metabolites against endothelial dysfunction in human aortic endothelial cells. Mol Nutr Food Res. 2015; 59: 2523–2536. 10.1002/mnfr.201500361 26390376

[59] Bjarnason I, MacPherson A, Hollander D. Intestinal permeability: an overview. Gastroenterology. 1995; 108: 1566–1581. 7729650 10.1016/0016-5085(95)90708-4

[60] Menard S, Cerf-Bensussan N, Heyman M. Multiple facets of intestinal permeability and epithelial handling of dietary antigens. Mucosal Immunol. 2010; 3: 247–259. 10.1038/mi.2010.5 20404811

[61] Blair SA, Kane SV, Clayburgh DR, Turner JR. Epithelial myosin light chain kinase expression and activity are upregulated in inflammatory bowel disease. Lab Invest. 2006; 86: 191–201. 10.1038/labinvest.3700373 16402035

[62] Laukoetter MG, Nava P, Nusrat A. Role of the intestinal barrier in inflammatory bowel disease. World J Gastroenterol. 2008; 14: 401–407. 10.3748/wjg.14.401 18200662 PMC2679128

[63] Pastorelli L, de Salvo C, Mercado JR, Vecchi M, Pizarro TT. Central role of the gut epithelial barrier in the pathogenesis of chronic intestinal inflammation: lessons learned from animal models and human genetics. Front Immunol. 2013; 4: 280 10.3389/fimmu.2013.00280 24062746 PMC3775315

[64] Corona G, Deiana M, Incani A, Vauzour D, Dessi MA, Spencer JPE. Inhibition of p38/CREB phosphorylation and COX-2 expression by olive oil polyphenols underlies their anti-proliferative effects. Biochem Biophys Res Commun. 2007; 362: 606–611. 10.1016/j.bbrc.2007.08.049 17727817

[65] Wells CL, Jechorek RP, Kinneberg KM, Debol SM, Erlandsen SL. The isoflavone genistein inhibits internalization of enteric bacteria by cultured Caco-2 and HT-29 enterocytes. J Nutr. 1999; 129: 634–640. 10082767 10.1093/jn/129.3.634

[66] Suzuki T, Hara H. Role of flavonoids in intestinal tight junction regulation. J Nutr Biochem. 2011; 22: 401–408. 10.1016/j.jnutbio.2010.08.001 21167699

[67] Piegholdt S, Pallauf K, Esatbeyoglu T, Speck N, Reiss K, Ruddigkeit L, et al Biochanin A and prunetin improve epithelial barrier function in intestinal CaCo-2 cells via downregulation of ERK, NF-kappaB, and tyrosine phosphorylation. Free Radic Biol Med. 2014; 70: 255–264. 10.1016/j.freeradbiomed.2014.02.025 24631489

[68] Turner JR. Intestinal mucosal barrier function in health and disease. Nat Rev Immunol. 2009; 9: 799–809. 10.1038/nri2653 19855405

[69] Lee SH. Intestinal permeability regulation by tight junction: implication on inflammatory bowel diseases. Intest Res. 2015; 13: 11–18. 10.5217/ir.2015.13.1.11 25691839 PMC4316216

[70] Suzuki T. Regulation of intestinal epithelial permeability by tight junctions. Cell Mol Life Sci. 2013; 70: 631–659. 10.1007/s00018-012-1070-x 22782113 PMC11113843

[71] Al-Sadi R, Ye D, Dokladny K, Ma TY. Mechanism of IL-1beta-induced increase in intestinal epithelial tight junction permeability. J Immunol. 2008; 180: 5653–5661. 18390750 10.4049/jimmunol.180.8.5653 PMC3035485

[72] Capaldo CT, Nusrat A. Cytokine regulation of tight junctions. Biochim Biophys Acta. 2009; 1788: 864–871. 10.1016/j.bbamem.2008.08.027 18952050 PMC2699410

[73] Wang F, Graham WV, Wang Y, Witkowski ED, Schwarz BT, Turner JR. Interferon-gamma and tumor necrosis factor-alpha synergize to induce intestinal epithelial barrier dysfunction by up-regulating myosin light chain kinase expression. Am J Pathol. 2005; 166: 409–419. 15681825 10.1016/s0002-9440(10)62264-x PMC1237049

[74] Ma TY, Boivin MA, Ye D, Pedram A, Said HM. Mechanism of TNF-{alpha} modulation of Caco-2 intestinal epithelial tight junction barrier: role of myosin light-chain kinase protein expression. Am J Physiol Gastrointest Liver Physiol. 2005; 288: G422–30. 10.1152/ajpgi.00412.2004 15701621

[75] Yap WH, Lim YM. Mechanistic Perspectives of Maslinic Acid in Targeting Inflammation. Biochem Res Int. 2015; 2015: 279356 10.1155/2015/279356 26491566 PMC4600485

[76] Rodriguez-Rodriguez R. Oleanolic acid and related triterpenoids from olives on vascular function: molecular mechanisms and therapeutic perspectives. Curr Med Chem. 2015; 22: 1414–1425. 25515513 10.2174/0929867322666141212122921

[77] Martin MA, Ramos S, Granado-Serrano AB, Rodriguez-Ramiro I, Trujillo M, Bravo L, et al Hydroxytyrosol induces antioxidant/detoxificant enzymes and Nrf2 translocation via extracellular regulated kinases and phosphatidylinositol-3-kinase/protein kinase B pathways in HepG2 cells. Mol Nutr Food Res. 2010; 54: 956–966. 10.1002/mnfr.200900159 20166143

[78] Tremaroli V, Backhed F. Functional interactions between the gut microbiota and host metabolism. Nature. 2012; 489: 242–249. 10.1038/nature11552 22972297

[79] Nicholson JK, Holmes E, Kinross J, Burcelin R, Gibson G, Jia W, et al Host-gut microbiota metabolic interactions. Science. 2012; 336: 1262–1267. 10.1126/science.1223813 22674330

[80] Cani PD, Osto M, Geurts L, Everard A. Involvement of gut microbiota in the development of low-grade inflammation and type 2 diabetes associated with obesity. Gut Microbes. 2012; 3: 279–288. 10.4161/gmic.19625 22572877 PMC3463487

[81] Cani PD. Crosstalk between the gut microbiota and the endocannabinoid system: impact on the gut barrier function and the adipose tissue. Clin Microbiol Infect. 2012; 18 Suppl 4: 50–53. 10.1111/j.1469-0691.2012.03866.x 22647050

[82] Frazier TH, DiBaise JK, McClain CJ. Gut microbiota, intestinal permeability, obesity-induced inflammation, and liver injury. JPEN J Parenter Enteral Nutr. 2011; 35: 14S–20S. 10.1177/0148607111413772 21807932

[83] Wang M, Donovan SM. Human microbiota-associated swine: current progress and future opportunities. ILAR J. 2015; 56: 63–73. 10.1093/ilar/ilv006 25991699 PMC7108572

[84] Heinritz SN, Mosenthin R, Weiss E. Use of pigs as a potential model for research into dietary modulation of the human gut microbiota. Nutr Res Rev. 2013; 26: 191–209. 10.1017/S0954422413000152 24134811

