# Supplementary figures and images for: Olive oil bioactives protect pigs against experimentally-induced chronic inflammation independently of alterations in gut microbiota
Source: PLoS One. 2017 Mar 27;12(3):e0174239. doi: 10.1371/journal.pone.0174239 (PMC5367713; doi:10.1371/journal.pone.0174239)

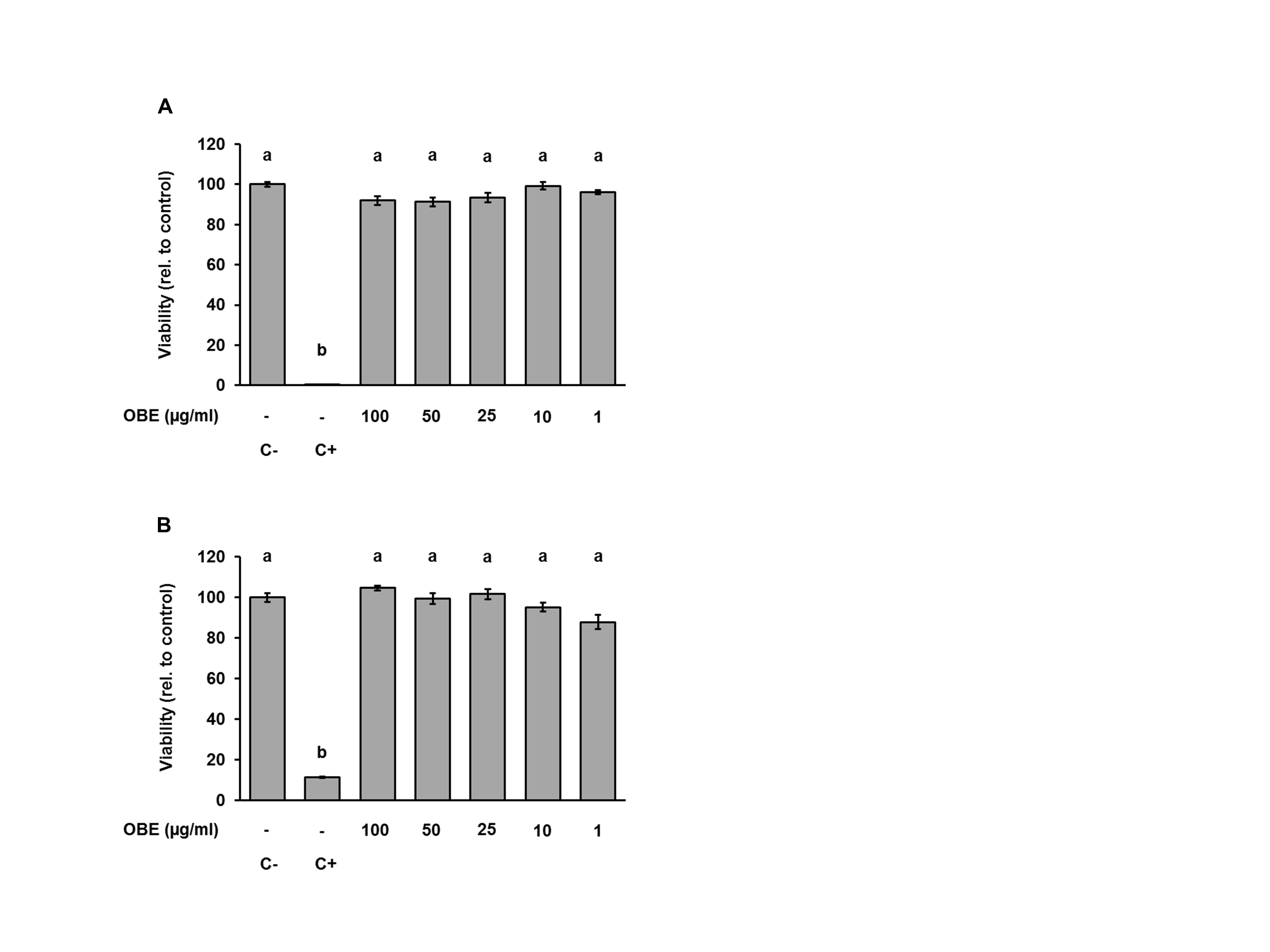

Supplement: S1 Fig — Cytotoxicity of OBE as assessed by neutral red assay and expressed as remaining cell viability after treatment. Cells were incubated with DMSO (0.1% v/v, C-), ethanol (10%, C+) or increasing concentrations of OBE. Bars represent means ± SEM of 2 independent experiments performed in triplicate. Different letters indicate significant differences among treatments (p < 0.05). (TIF) [file pone.0174239.s001.tif]
